# 

**Published:** 1861-01

**Authors:** 


### CONTENTS OF VOLUME XVI.

PAGE

Amaurosis caused by a Carious Tooth.... 14
A Practical Treatise on Mechanical Dentistry. By J. Richardson, D. D. S., M. D.... 16
A Plea for Beard.... 54
"A. B.".... 63
Argentine.... 63
A Retrospective and Prospective View of Dentistry By W. A. Pease.... 69
Advice Wanted.... 111
Another death from Chloroform.... 122
Anæsthetics. By John Snow. M. D.... 130
A Cork Vise.... 132
Address to the Graduating Class of the Ohio College of Dental Surgery, Session 1860-61. By J Richardson.... 133
A Letter from Philadelphia.... 190, 2[illegible]2
Alveolar abscess—treatment and cure. By W. H. Shadoon.... 221
A New Napkin Holder. By W A. Pease. 225
A Practical Treatise on Phthisis Pulmonalis, etc.... 260
Actions of different Medicines on the Mental Faculties.... 318
Another Lock Jaw Case.... 371
A new Anæsthetic.... 380
A new Dental College.... 388
A Drill Shield.... 386
A Mill for the Toothless.... 402
Anæsthetic.... 405
American Agriculturist.... 484
Aluminum in Greenland.... 477
A new Anæsthetic—Kerosolene.... 570
A Model Patient.... 580
Alloys for Die-Metal. By Dr. B. Wood. 582
American Dental Convention.... 590
A Course of Six Lectures on the Chemical History of a Candle, etc.... 671
American Journal of Dental Science.... 666
Associations.... 670

Bouchet's Pluggers.... 64
Block Teeth.... 239
Brown on Secretory Influences.... 373
Bleaching.... 376

Calcium and its Compounds.... 629
Concerning Dental Literature, etc.... 641
Chloride of Zinc Moulded into Sticks for the purpose of Cauterization.... 51
Commencement Exercises.... 129
Central Ohio Dental Association.... 165
Causes which modify the action of Anæsthetics.... 180
Chloroform.... 418
Closure of the Parotid Duct.... 483–434
Credit.... 433
Correction.... 433
Concerning Tooth Extraction. By J. A. McClelland.... 457
Convulsion from Dentition.... 620
Causes which retard Dental Progress.... 613
Case for Office Lathe.... 668
Close of the Volume.... 667

PAGE

Dangers incident to Anæsthetics.... 180
Dental Machinery.... 194
Dental Exhibitions. By J. Taft.... 10
Death from Chloroform.... 57
Does Amalgam Shrink ?.... 131
Dentition.... 147
Dentist's Memorandum for 1861.... 672
Dental Fees.... 151
Dr. Chas. Bonsall.... 196
Dental Patents.... 255
Diagnosis.... 282
Death from Anæsthesia.... 302
Diversities of Dental Practice, by J. Taft. 391
Dental Depot.... 627
Dental Empiricism—past and present.... 407
Dentists in the Army.... 430
Dr. J. Allen's Review of Who are Dentists 403
Dentition and its Derangements, 469, 597, 654
Dissolution of the Union.... 483
Disease of the Mouth.... 516
Dental Hospitals.... 531
Diseased Antrum.... 579
Dentistry in California.... 662

Extensive Medullary Cancer of the Antrum.... 187
Editorial. 195, 129, 60, 255, 320, 383, 430, 479, 529, 575-6, 666
Extracts from the Preceedings of the Wayne County Medical Society.... 318
Epithelial Cancer of the Lip.... 306
Excursion to Chicago.... 482
Extraction.... 532
Exostosis and Inflammatory Tumors.... 522

Fifteenth Volume.... 61
Fracture of the Lower Jaw.... 52
Fermentation of Milk in the Mammæ.... 49
Formation of Local Societies.... 226
Forceps.... 259
Free Nation.... 256
Facial Neuralgia, the result of Chronic Rheumatism.... 112
First Dentition.... 12
Fibro,melanoid Tumor of the Antrum, etc 99
Filling Teeth. By W. M. Rogers.... 325
Fistulous Opening through the Cheek.... 48
"Filling Molar Teeth—Approximal Surfaces".... 479
Fusible Gauge.... 622

Gibson House.... 628
Gold. By Prof. W. Calvert.... 5
Gold.... 60

Human Knowledge, natural and supernatural.... 195
How to remove Amalgam Fillings.... 126
Human Knowledge and Impartation.... 386

Indiana State Dental Association—Notice 664
Iodine.... 196
Inflammation. By W. H. Atkinson..338–437
In all thy ways acknowledge God.... 388
Internal Decay....

PAGE

Influence of Association. By Dr. J. G. Hamill ........................ 442
Impartation ........................ 261
Indiana State Dental Association........ 399
Improvement in Rubber Plates.......... 649

"Just to please his Mother,"........... 13

Kentucky State Dental Assoc'.n..38, 297, 340

Labor........................ 533
Land Scenery........................ 427
Lancing the Gums........................ 323
Large Fillings........................ 321
Life and Character of Chapin A. Harris, M. D., D. D. S.................. 77
Lost, Strayed or Stolen ................ 258
Letters from New York................ 29
" " Philadelphia................ 37

Mastication and Articulation of Artificial Dentures ........................ 616
Microscopic.................... 434, 484
Microscope........................ 432
Michigan Dental Association............ 160
Minutes of the Ninth Annual Meeting of the Ohio Dental College Association. 168
Minutes of the Seventeenth Annual Meeting of the Miss. Valley Association of Dental Surgeons............... 129, 174
Minutes of the Cincinnati Local Dental Association.................. 98, 27
Mad River Valley Society............... 254
Meeting........................ 671

Napkin Holder........................ 257
New Dental Society.................... 132
New Caustic Holder.................... 385
Notices........................ 429
New Anæsthetics, etc.................. 572
New Method of Producing Local Anæsthesia........................ 53
New Application of Chloroform in Neuralgia and in certain Rheumatic Complaints........................ 43

Oxygen as an Antidote to Asphyxia from Chloroform and Ether.............. 506
On the Illumination of the Cavities of the Body by means of Electricity....... 114
On the use of Looped Wire in the removal of Foreign Bodies from the Air Passages, etc........................ 109
On Chloroform and the Narcotic Remedial Remedies, etc...................... 308
Our Ignorance........................ 195
On a Method of Obtaining Homogeneous Light of great intensity............. 428
Obituaries, By A. Berry............... 15

Plates over Fangs...................... 213
Professional Success. By A. S. Talbert, D. D. S., Lexington................ 276

PAGE

Personal.................... 667, 387, 578, 628
Professional Advancement. By Dr. S. Clippenger........................ 369
Postponement.................... 383, 575
Practical Hints........................ 403
Patriotic ........................ 628
Pretty Fast........................ 132
Pressure as a Preventive of Inflammation 59

Report of Discussions.................. 388
Review of Who are Dentists. By J. Allen, D. D. S..................... 585
Rubber Work........................ 624
Reply. By A. Evans.................. 144
Refitting Temporary Plates. By J. Richardson........................ 156
Remarks on the nature of the changes which occur after the Extraction of the Natural Teeth, and the propriety of the early introduction of Artificial Substitutes. By Prof. J. Richardson. 91
Rubber Work. By Geo. L. Paine, D.D,S. 94
Remarks on Dentistry in the Army...... 525
Render unto Cæsar.................. 63
Remarks on the Use of Tobacco......... 660

Salivary Glands in the Breasts ......... 64
Stramonium in Neuralgia................ 317
Seventeenth Annual Meeting of the Miss. Valley Association .................. 227

The Annoyances of the Administration of Chloroform ........................ 307
The Dentist's Memorandum.............. 131
The Ohio Dental College Association.... 130
The Pocket Anatomist.................. 68
The Hippotamus with the Toothache.... 67
The Extraction of Teeth ............... 243
The New Anæsthetic—Keroselene...... 621
Temporal Economy..................... 575
The Beard Question................... 379
The Surgical Diseases of the Tongue..... 381
The Amalgam Question......... 389, 451, 485
The American Dental Convention....... 479
Tricks of the Type.................... 578
Treatment of Abscess.................. 625
The Preliminary Education of Medical Students ........................ 650
Teeth........................ 666
The Physician's Pocket Memorandum for 1862........................ 672

Vulcanite Work........................ 440

"Wholly Given to Idolatry".......... 387
Wood for Finishing Fillings............. 580
Who are Dentists. By Wm. A. Pease... 365
153, 291, 216, 492
Water—Its History, Characteristics, etc. 507
604
Working in Aluminum.................. 503

